# Real-Time Pose Measurement Framework of Wind Tunnel Aircraft Models Based on a Monocular Time-of-Flight Camera

**DOI:** 10.3390/s26051476

**Published:** 2026-02-26

**Authors:** Jianqiang Huang, Cui Liang, Shuai Zhao, Tengchao Huang

**Affiliations:** 1 College of Optical Science and Engineering, Zhejiang University, Hangzhou 310027, China; j.q.huang@zju.edu.cn (J.H.); cui_liang@zju.edu.cn (C.L.); 0623868@zju.edu.en (S.Z.); 2 Jiaxing Research Institute, Zhejiang University, Jiaxing 314000, China; 3 Shanghai Institute for Advanced Study, Zhejiang University, Shanghai 201203, China

**Keywords:** point cloud registration, pose estimation, time-of-flight (ToF) camera, wind tunnel testing, monocular 3D sensing, non-contact optical measurement

## Abstract

Precise and real-time acquisition of aircraft model attitude is fundamental for aerodynamic analysis in wind tunnel experiments, yet achieving high-precision non-contact measurement remains a significant challenge. To address this, this paper proposes a pose measurement framework based on a monocular Time-of-Flight (ToF) camera that fuses keyframe global registration with non-keyframe local registration. First, a novel hand-crafted local feature based on three-plane encoded height and density is introduced. When combined with the Two-stage Consensus Filtering RANSAC (TCF-RANSAC) algorithm, this feature achieves robust global registration of keyframes, providing reliable initial pose estimates for the system. Subsequently, leveraging the continuity constraint of model motion, fast incremental local registration of non-keyframes is performed using the Generalized Iterative Closest Point (GICP) algorithm, which avoids falling into local optima while significantly improving computational efficiency. Evaluation results on simulated datasets with synthetic noise and a real experimental platform demonstrate that the method achieves a single-axis rotation angle error of less than $0.03^{\circ }$ while processing at over 40 FPS, satisfying real-time measurement requirements. Comparative evaluations against multiple existing registration methods indicate that the proposed framework achieves superior accuracy and robustness, reducing rotation angle errors by 9% to 39% compared to mainstream global registration methods under single-view ToF sensing conditions. Furthermore, this study quantifies the error distribution characteristics of monocular ToF-based pose estimation, revealing an “axis-sensitivity” phenomenon where the rotation error around the optical axis is significantly lower (e.g., $0.02^{\circ }$, $0.03^{\circ }$) than that around the orthogonal axes (e.g., $0.38^{\circ }$, $0.26^{\circ }$). These findings provide practical guidance for camera placement and system design in high-precision aerodynamic measurement scenarios.

## 1. Introduction

Wind tunnel experiments involving aircraft [1,2], automobiles, and other targets [3,4] are pivotal for equipment development in the aerospace and transportation sectors. By synchronizing timestamps, it is possible to obtain the pose, pressure, and other physical parameters of different targets simultaneously. Therefore, achieving fast and accurate target pose measurement is of significant importance [5].

Traditional pose measurement methods can be categorized into two types: contact-based and non-contact. Contact-based methods typically implement pose measurement by rigidly attaching inertial measurement units (IMUs) to the target [6]. Although these methods provide high-rate attitude outputs with relatively low algorithmic complexity, they require additional experimental setup and suffer from cumulative drift, which limits long duration accuracy. In contrast, non-contact methods acquire target geometry using optical devices, such as RGB cameras, laser-based vision systems, and Time-of-Flight (ToF) cameras, and estimate pose through image or point cloud processing algorithms [7,8,9]. Marker-based vision systems are widely used in wind tunnel experiments due to their high accuracy and maturity, but they require careful marker placement and are sensitive to occlusion at large angles of attack. Markerless vision-based approaches reduce surface preparation requirements but are sensitive to illumination changes, background interference, and partial occlusion; moreover, monocular RGB-based methods provide only indirect depth cues, limiting achievable three-dimensional pose accuracy. According to reported wind tunnel experiments, conventional aerodynamic measurements generally require attitude accuracy on the order of 0.05–0.1°, while advanced aeroelastic and flow–structure interaction studies demand higher precision, typically better than 0.02–0.03°, with update rates exceeding 30–50 Hz to ensure reliable synchronization with other sensors [1,3,4], which poses significant challenges for existing inertial-based and vision-based methods.

Point clouds are a widely used 3D data representation in computer vision and graphics, typically acquired from devices such as LiDAR, ToF cameras, and structured light cameras [10,11,12]. Focusing on pose measurement scenarios for wind tunnel models, two key considerations emerge. First, wind tunnels are typically large-scale environments, requiring 3D imaging devices with sufficient working distance that maintain consistent precision across varying ranges. Second, to achieve temporal alignment with other sensor data, algorithms must enable real-time pose measurement, necessitating devices capable of high-speed point cloud acquisition. Considering these requirements, structured light systems are limited by light source power and constrained working distances; while capable of sub-millimeter precision, they operate slowly and struggle with fast-moving targets. Compared to structured light, LiDAR achieves significantly greater working distances but typically offers only centimeter-level precision and near-real-time scanning. However, ToF cameras typically achieve an 8–10 m working distance with millimeter-level precision while enabling real-time point cloud acquisition, making them an optimal choice for this application. Table 1 summarizes this comparison.

Through point cloud registration, targets from multiple coordinate systems can be aligned to a unified frame to determine camera or target pose changes. Early representative work includes the Iterative Closest Point (ICP) algorithm [13] and its variants [14,15,16]. To improve registration precision and robustness, Segal et al. proposed the Generalized ICP (GICP) algorithm [17]. GICP unifies point-to-point and point-to-plane metrics by introducing covariance matrices for each point to model local surface geometry, essentially implementing a plane-to-plane probabilistic registration framework that significantly improves performance in structured environments. However, both ICP and GICP are local algorithms and typically require good initial alignment to converge to the correct solution. To overcome this, the Go-ICP algorithm employs branch-and-bound methods for global search but suffers from low computational efficiency [18]. Methods based on hand-crafted local feature descriptors like SHOT [19], TOLDI [20], and FPFH [21] can provide good initializations for ICP and GICP, serving as widely used coarse registration approaches that avoid local optima. Nevertheless, hand-crafted descriptors rely on human intuition and consequently suffer from inherent limitations in feature representation capability [22,23].

Recent years have witnessed significant progress in deep learning-based feature description and point cloud registration [24,25,26]. Methods such as SpinNet [27] and Geo-Transformer [28] achieve robust registration in large-scale indoor and outdoor scenes, substantially improving feature representation. However, deep learning-based methods still face challenges, including high memory usage, substantial computational overhead, and the requirement for large training datasets. These issues impede their practical deployment in wind tunnel experimental scenarios.

Whether hand-crafted or deep learning-based, descriptor-based registration methods inevitably produce numerous false matches due to limited local feature distinctiveness, noise, and repetitive structures. Therefore, it is necessary to filter for high-quality correspondences to provide a reliable initial alignment for fine registration methods like ICP [29,30]. The maximal clique method relaxes the constraints of previous graph-based approaches, achieving significant progress in improving inlier rates, and has been applied to various scenarios [31,32]. However, this method is sensitive to the number of initial correspondences, and graph construction is time-consuming, limiting its utility in time-critical applications. RANSAC is the most common method for handling outliers [33], generating and validating hypotheses through repeated sampling. However, the required number of iterations grows exponentially with the outlier ratio, rendering it significantly less efficient than ideal. The recently proposed Two-stage Consensus Filtering RANSAC (TCF-RANSAC) applies multi-level filtering, substantially improving computational efficiency while maintaining high accuracy, thus enabling real-time applications [34].

Previous work on point cloud-based pose measurement has often treated two consecutive frames as independent datasets, neglecting model motion constraints [8]. In wind tunnel trials, models typically do not undergo abrupt large-scale motion over short periods. Therefore, this paper addresses the pose measurement problem for aircraft in wind tunnels by first analyzing pose representation across multiple coordinate systems and then proposing a framework that fuses keyframe global registration with local registration. For keyframe global registration, a novel hand-crafted local feature is utilized; initial correspondences obtained through feature matching are refined using TCF-RANSAC for robust estimation, followed by fine-tuning with the GICP algorithm. For non-keyframe local registration, incremental pose updates are implemented by using the pose output from the previous frame as the initialization for the current frame, thereby bypassing the computational burden of global registration. Finally, the proposed method demonstrates high accuracy and efficiency when evaluated on both simulated and real datasets.

We select the hand-crafted local descriptor + TCF-RANSAC + GICP combination mainly to satisfy real-time requirements and practical deployment constraints in single-view ToF wind tunnel measurements. Learning-based descriptors and registration frameworks can achieve strong representation, but they typically require training data, incur higher memory/compute cost, and may generalize poorly to ToF point clouds with sensor-specific noise, sparse sampling, and limited viewpoint coverage. In contrast, a hand-crafted descriptor is training-free and deterministic, which facilitates repeatable experiments and rapid integration. TCF-RANSAC is adopted for correspondence filtering because it improves hypothesis verification efficiency under high outlier ratios while maintaining robustness, which is important when smooth surfaces and ToF noise reduce descriptor distinctiveness. Finally, GICP is used for fine registration because its covariance-based formulation provides a probabilistic plane-to-plane metric that is robust to anisotropic ToF ranging noise and yields accurate refinement with reasonable initialization.

The framework is intended for wind tunnel experiments requiring non-contact, full six-degree-of-freedom pose measurement of rigid aircraft models, such as attitude monitoring during angle-of-attack sweeps and aerodynamic force and moment measurements where fiducial markers or onboard sensors are undesirable. Its practical applicability is constrained by the intrinsic parameters of the employed monocular ToF camera, including working distance, field of view, spatial resolution, and ranging accuracy, and it requires the model to remain within the camera’s field of view to ensure sufficient point cloud overlap. The method is not limited to static pose estimation; by combining keyframe-based global registration with efficient local registration on subsequent frames, it supports real-time continuous pose tracking of smoothly moving models, provided that abrupt large-scale motion and severe self-occlusion are avoided.

The main contributions of this work are summarized as follows:

1. A complete real-time pose measurement pipeline: We propose a comprehensive framework fusing keyframe global registration (using a novel three-plane encoded feature and TCF-RANSAC) with incremental local updates (GICP), achieving a rotation error of less than 0.03° at speeds exceeding 40 FPS.
2. Characterization of sensing limitations: The error distribution of monocular ToF measurement is systematically analyzed, revealing an “axis-sensitivity” phenomenon where optical axis rotation offers superior precision. This finding provides essential theoretical guidelines for optimal camera deployment in aerodynamic testing.

## 2. Problem Formulation

Let the model point cloud acquired by the camera at time 0 be $P_{C,0}$ and at time *t* be $P_{C,t}$, both represented in the camera coordinate system *C*. The objective is to estimate the rigid body transformation $T_{reg}\in \mathrm{SE}\left(3\right)$ (with rotation R∈SO(3) and translation $t\in R^{3}$) to obtain the model pose change in the camera coordinate system by rigid registration. Subsequently, the estimated pose in *C* is converted to an equivalent pose representation in the model coordinate system *M* or the wind tunnel/world coordinate system *W*.

This section outlines the theoretical foundations of the proposed method. First, the projection geometry and data acquisition characteristics of the sensor are modeled to define the relationship between 3D spatial points and the image plane. Subsequently, the mathematical principles governing the alignment of 3D point clouds are detailed to formulate the core registration objective. Finally, the spatial relationships between the camera, the model, and the mechanism are formalized through coordinate system transformations, deriving the necessary geometric constraints for accurate pose estimation and error evaluation.

### 2.1. ToF Camera Model

**Figure 1:**
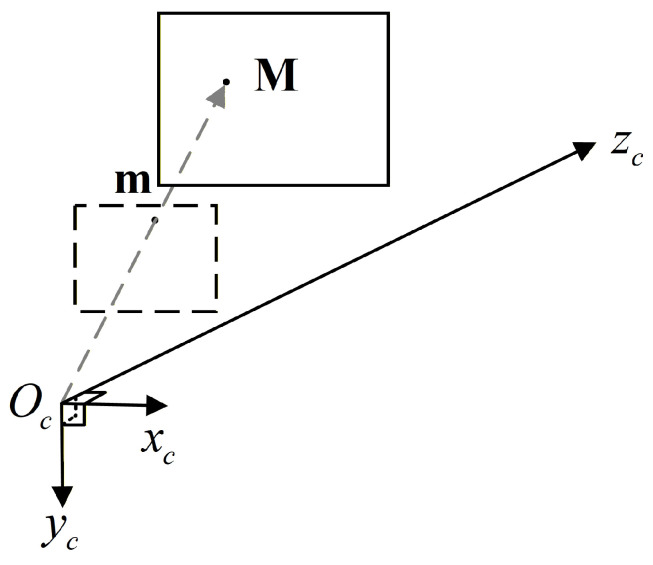
ToF camera model, where $O_{c}$ is the origin of the camera coordinate system, and $x_{c}$, $y_{c}$, and $z_{c}$ are three orthogonal axes in the camera coordinate system.

The process of point cloud acquisition by a ToF camera can be described using the pinhole camera model. As shown in Figure 1, let a 3D point in the world coordinate system be represented as $M={\left[X_{M},Y_{M},Z_{M}\right]}^{T}$ and its projection point on the pixel plane be $m={\left[u,v\right]}^{T}$. The imaging model can be expressed as follows:

(1) $$d\left[\begin{array}{c} u\\ v\\ 1 \end{array}\right]=K\left[\begin{array}{c} X_{M}\\ Y_{M}\\ Z_{M} \end{array}\right],$$

where K is the camera intrinsic matrix, defined as

(2) $$K=\left[\begin{array}{ccc} f_{x} & 0 & c_{x}\\ 0 & f_{y} & c_{y}\\ 0 & 0 & 1 \end{array}\right],$$

where $f_{x}$ and $f_{y}$ are focal lengths in the *x* and *y* directions, respectively, $c_{x}$ and $c_{y}$ are the principal point, and *d* is the depth measured by the ToF camera.

### 2.2. Point Cloud Registration

A point cloud is a collection of 3D spatial coordinates. We denote the source point cloud as P and the target point cloud as Q. The objective of rigid point cloud registration is to find a homogeneous transformation T that minimizes the alignment error between P and Q [13]:

(3) $$\min_{R,t}\sum_{(p_{i},q_{i})\in C}∥Tp_{i}-q_{i}{∥}^{2}=\min_{R,t}\sum_{(p_{i},q_{i})\in C}{∥Rp_{i}+t-q_{i}∥}^{2}$$

where R∈SO(3) is a 3D rotation matrix, t is a 3D translation vector, and C is the set of matched point pairs, where each pair $\left(p_{i},q_{i}\right)$ consists of a source point $p_{i}\in P$ and a target point $q_{i}\in Q$. The homogeneous transformation T can be written as

(4) $$T=\left[\begin{array}{cc} R & t\\ 0 & 1 \end{array}\right].$$

Furthermore, R can be written as

(5) $$R=\left[\begin{array}{ccc} r_{11} & r_{12} & r_{13}\\ r_{21} & r_{22} & r_{23}\\ r_{31} & r_{32} & r_{33} \end{array}\right].$$

In most cases, C is unknown. Within the ICP framework, it is typically assumed that the nearest point in Q to a point in P forms a matched pair, with iterations yielding R and t that minimize residuals. However, this assumption makes the algorithm susceptible to initial values and falling into local optima. A good solution is to use initial matched points and the initial transformation obtained through feature matching as initial values to improve robustness.

### 2.3. Coordinate System Definition and Transformation

**Figure 2:**
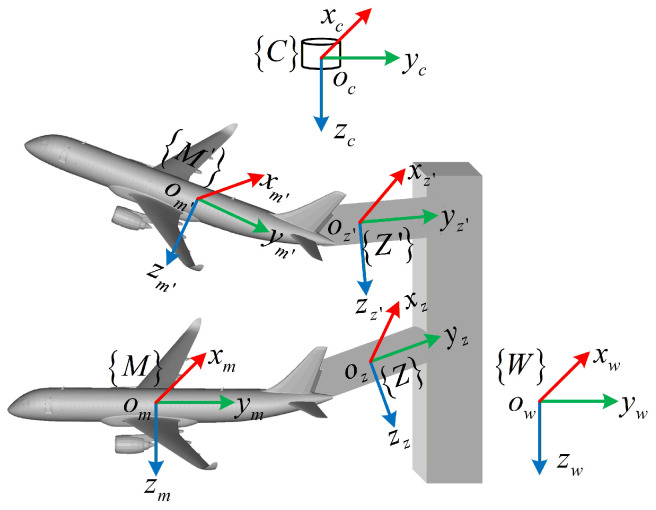
Relevant coordinate systems in the measurement scenario: W denotes the wind tunnel coordinate system, C denotes the camera coordinate system, M and M′ denote the model coordinate systems before and after motion, and Z and Z′ denote the mechanism coordinate systems before and after motion. $x_{c}$, $x_{m}$, $x_{z}$, and $x_{w}$, respectively, denote the *x*-axes of the camera, model, mechanism, and world coordinate systems, with similar interpretations for the *y* and *z* axes.

Figure 2 illustrates the coordinate system definitions for ToF camera-based pose measurement of aircraft. The model coordinate system *M* and mechanism coordinate system *Z* are moving, while the camera coordinate system *C* and world coordinate system *W* are stationary. We adopt the convention that the transformation from coordinate system *C* to coordinate system *M* is denoted as $T_{C}^{M}$. Since the model is rigidly attached to the mechanism, the transformation $T_{M}^{Z}$ from *M* to *Z* remains constant during measurement. In the wind tunnel scenario, *M* is fixed to the aircraft body as the visual observation target, while *Z* is fixed to the mechanical support providing the kinematic reference; thus, the coordinate systems before and after motion refer to the temporal states of these rigidly coupled systems at the initial time and the current measurement time *t*.

Let the model point cloud acquired by the camera at time 0 be $P_{C,0}$ and at time *t* be $P_{C,t}$. Then,

(6) $$P_{C,t}=T_{reg}\;\cdot \;P_{C,0},$$

where $T_{reg}$ is the transformation matrix from the point cloud at time 0 to time *t*.

For the same physical point on the model, its coordinates in the model coordinate system remain invariant:

(7) $$P_{M}=T_{C}^{M}\;\cdot \;P_{C,0}=T_{C}^{M^{\prime }}\;\cdot \;P_{C,t},$$

Therefore,

(8) $$T_{reg}={\left(T_{C}^{M^{\prime }}\right)}^{-1}\;\cdot \;T_{C}^{M},$$

where $T_{C}^{M}$ is the transformation matrix from the camera coordinate system to the model coordinate system at time 0. The transformation from model coordinate system $M^{\prime }$ at time *t* to model coordinate system *M* at time 0, denoted $T_{M^{\prime }}^{M}$, is as follows:

(9) $$T_{M^{\prime }}^{M}=T_{C}^{M}\;\cdot \;T_{M^{\prime }}^{C}=T_{C}^{M}\;\cdot \;T_{reg}\;\cdot \;T_{M}^{C}.$$

Clearly, $T_{M^{\prime }}^{M}$ and $T_{reg}$ are similar. Furthermore, $T_{M^{\prime }}^{M}$, $T_{reg}$, and $T_{Z^{\prime }}^{Z}$ form a complete relational network through similarity transformation, describing the same physical motion represented in different coordinate systems.

When evaluating the accuracy of $T_{reg}$ using the mechanism’s pose transformation $T_{Z^{\prime }}^{Z}$,

(10) $$T_{Z^{\prime }}^{Z}=T_{C}^{Z}\;\cdot \;T_{reg}\;\cdot \;T_{Z}^{C},$$

which also implies the following:

(11) $$T_{Z^{\prime }}^{Z}=T_{M}^{Z}\;\cdot \;T_{C}^{M}\;\cdot \;T_{reg}\;\cdot \;T_{M}^{C}\;\cdot \;T_{M}^{Z},$$

Similarly, the model’s pose transformation in the wind tunnel coordinate system $T_{W}$ also satisfies the following:

(12) $$T_{W}=T_{C}^{W}\;\cdot \;T_{reg}\;\cdot \;T_{W}^{C}.$$

## 3. Methodology

**Figure 3:**
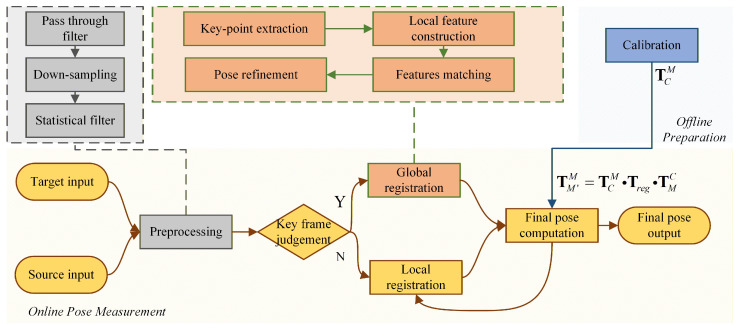
Pose measurement pipeline fusing keyframe global registration with non-keyframe local registration.

Figure 3 illustrates the proposed framework, which fuses keyframe global registration with incremental local tracking. To balance computational efficiency and robustness against local optima, the system processes the initial frame (keyframe) using a global registration pipeline to establish a reliable pose baseline. Subsequent frames (non-keyframes) are then aligned using fast local registration, initialized by the previous time-step’s pose. This strategy leverages the motion continuity of wind tunnel models to achieve real-time performance. The detailed procedure is outlined below and summarized in Algorithm 1.
**Algorithm 1** Proposed pose measurement pipeline.**Input:** Current point cloud $P_{C,t}$; Reference point cloud $P_{C,0}$;    Previous registration result $T_{reg,t-1}$ (if t>0); Calibration $T_{C}^{M}$.**Output:** Model pose change $T_{M^{\prime }}^{M}$ (in Model coordinate system).1: **Preprocessing:** Apply pass-through filter, grid downsampling, and statistical outlier removal  on $P_{C,t}$ (Section 3.1).2: **Registration (Obtain $T_{reg}$):**    **If** *t* is a Keyframe **then** (Global Registration + Refinement)        2.1: Extract keypoints and construct local features on $P_{C,t}$ and $P_{C,0}$.        2.2: Perform feature matching and apply **TCF-RANSAC** to get coarse pose $T_{coarse}$.        2.3: **Pose Refinement:** Apply **GICP** initialized with $T_{coarse}$ to minimize:          $J\left(T\right)=\sum_{i}d_{i}^{⊤}{\left(C_{i}^{P_{C,0}}+TC_{i}^{P_{C,t}}T^{⊤}\right)}^{-1}d_{i}$ (Equation (22)).        2.4: Update $T_{reg}\leftarrow T_{optimized}$.    **else** (Local Registration)        2.5: Set initialization $T_{init}\leftarrow T_{reg,t-1}$ (Motion Continuity).        2.6: **Tracking:** Apply **GICP** initialized with $T_{init}$ to solve for $T_{reg}$.    **end if**3: **Final Pose Computation:**    3.1: Convert pose to Model coordinate system using Equation (9):      $T_{M^{\prime }}^{M}=T_{C}^{M}\;\cdot \;T_{reg}\;\cdot \;{\left(T_{C}^{M}\right)}^{-1}$.4: **Output:** Return $T_{M^{\prime }}^{M}$.

### 3.1. Preprocessing

In the preprocessing step, the point cloud is $P=\{p_{i}\},i=1,2,\ldots ,N$, where *N* is the number of source points. We first remove invalid points through a pass-through filter:

(13) $$P_{pass}=\left\{p_{i}\left(x,y,z\right)\in P|x_{min}\leq x\leq x_{max},\;y_{min}\leq y\leq y_{max},\;z_{min}\leq z\leq z_{max}\right\},$$

where P is the original point cloud set, $P_{pass}$ is the filtered point cloud set, and $x_{min}$, $x_{max}$, $y_{min}$, $y_{max}$, $z_{min}$, and $z_{max}$ are user-defined minimum and maximum thresholds along the *x*-, *y*-, and *z*-axes, respectively.

Subsequently, we apply grid downsampling to reduce the point cloud size and apply statistical filtering to further remove point cloud noise.

### 3.2. Global Registration

#### 3.2.1. Keypoint Extraction

Keypoints, as a subset of the point cloud, are extracted for constructing local features. Keypoint selection methods are numerous [35,36]. Here, instead of deliberately selecting keypoints, we randomly select a fixed proportion to avoid having too many or too few keypoints.

#### 3.2.2. Local Feature Construction

We employ a previously proposed hand-crafted local feature construction method based on three-plane encoded height and density [22]. The construction steps are briefly described as follows:

For point cloud P and an extracted keypoint $p_{k}$ on P, we first construct a local reference frame (LRF) within a spherical neighborhood of radius *r* around $p_{k}$ to achieve rigidity invariance of local features [37].

Specifically, given the neighborhood point set P, the *z*-axis of the LRF is obtained as the eigenvector corresponding to the smallest eigenvalue of the covariance matrix of the de-centered neighborhood points. The sign ambiguity of the *z*-axis is resolved by enforcing consistency with the dominant direction of the neighborhood.

Subsequently, all neighborhood points are projected onto the plane orthogonal to the *z*-axis. On this projected point cloud, local height information along the *z*-axis is encoded and fused with planar distance information to construct a second covariance matrix, from which the *x*-axis is determined after sign disambiguation. Finally, the *y*-axis is computed as the cross product of the *x*-axis and the *z*-axis, forming a right-handed orthonormal basis centered at $p_{k}$.

The neighborhood point cloud $P^{\prime }=\left\{p_{i}^{\prime }\in R^{3}|i=1,2,\ldots ,K\right\}$ at $p_{k}$ yields an LRF:

(14) $$\mathrm{LRF}=\{x\left(p_{k}\right),y\left(p_{k}\right),z\left(p_{k}\right)\},$$

where *K* is the number of points in the neighborhood, and $x\left(p_{k}\right),y\left(p_{k}\right),z\left(p_{k}\right)$ form an orthonormal basis centered at keypoint $p_{k}$.

Subsequently, we transform neighborhood points to the local reference frame to obtain $P^{t}=\left\{p_{i}^{t}\in R^{3}|i=1,2,\ldots ,K\right\}$. We extract local features from three orthogonal projection planes of the LRF. Taking the plane $L_{xoy}=x+y=0$ as an example, we partition the plane into $M_{a}$ angular sectors and $N_{r}$ radial rings. For each region, we compute average density $\bar{d}$ and average height $\bar{h}$:

(15) $$\begin{array}{cc} \bar{d} & =\frac{1}{s}\sum_{i=1}^{s}∥p_{i}^{t}-c∥,\\ \bar{h} & =\frac{1}{s}\sum_{i=1}^{s}h_{i}, \end{array}$$

where *s* is the number of points in the region, *c* is the centroid of points in the region, and $h_{i}$ is the local height, defined as the z-component of points in the region.

Similar to the existing literature, we further obtain a Gaussian-encoded average density:

(16) $$g\left(\bar{d}\right)=\exp\left(\frac{-{\left({\bar{d}}_{min}-\bar{d}\right)}^{2}}{2\sigma^{2}\left({\bar{d}}_{max}-{\bar{d}}_{min}\right)}\right),$$

where σ is a bandwidth control parameter, and ${\bar{d}}_{max}$ and ${\bar{d}}_{min}$ are the maximum and minimum average densities across all regions, respectively. Average height is treated similarly. Additionally, we quantize the Gaussian-encoded average density:

(17) $$f\left(\bar{d}\right)=\frac{\log(1+\left(2\mu -1\right)\;\cdot \;g\left(\bar{d}\right))}{\log(1+(2\mu -1))},$$

where μ is the quantization bit depth. Average height is handled similarly. Finally, we concatenate the quantized average densities and quantized average heights from all partitions on the three planes to form the local feature *F*.

#### 3.2.3. Feature Matching

After obtaining local features for all keypoints, we use kdtree to find nearest neighbor matches between source and model point clouds, constructing the initial correspondence set C:

(18) $$C=\{c_{k}=\left(p_{k},q_{k}\right),k=1,2,\ldots ,N_{c}\},$$

where $p_{k}$ and $q_{k}$ are keypoints on the source and model point clouds, respectively, and $N_{c}$ is the number of initial correspondences.

However, due to ToF camera point cloud noise and limited viewpoint causing self-occlusion, the constructed local features have insufficient representation capability. Consequently, initial correspondences often contain many false matches requiring further filtering.

We use TCF-RANSAC to perform robust estimation on initial correspondences. TCF-RANSAC comprises three stages of progressive filtering. In the first stage, we randomly select a correspondence pair $c_{k}$ and iteratively construct a maximum consensus set $C^{\prime }$:

(19) $$\begin{array}{cc} C_{i}^{\prime } & =\{c_{j}|c_{j}\in C,\forall j,d\left(c_{j},c_{k}\right)<\tau \},\\ d(c_{j},c_{k}) & =\;{∥p_{j}-p_{k}∥}_{2}-{∥q_{j}-q_{k}∥}_{2}, \end{array}$$

where $C_{i}^{\prime }$ is a subset of C, *i* is the current iteration, ${∥\;\cdot \;∥}_{2}$ is the L2 norm, and τ is the distance constraint threshold. Note that for any correspondence $c_{x}\in C$, we denote its constituent points in the source and target clouds as $p_{x}$ and $q_{x}$, respectively.

In the second stage, building upon the first stage, we further introduce angle constraints. From $C^{\prime }$, we randomly select two correspondences $c_{i}$ and $c_{j}$, first satisfying the following equation:

(20) $$S_{ij}=\left\{c_{k}|c_{k}\in C^{\prime },\forall k,d\left(c_{k},c_{i}\right)<\tau ,d\left(c_{k},c_{j}\right)<\tau \right\},$$

Then, we iteratively construct a more robust consensus set $C^{\prime \prime }$:

(21) $$\begin{array}{cc} C_{i}^{\prime \prime } & =\{c_{m}\in S_{ij}|\alpha \left(c_{m},c_{i},c_{j}\right)<\beta \},\\ \alpha (c_{m},c_{i},c_{j}) & =∡p_{i}p_{m}q_{i}+∡p_{j}p_{m}q_{j},\\ \beta & =\left|\arcsin\frac{\tau }{∥p_{m}-p_{i}{∥}_{2}}+\arcsin\frac{\tau }{∥p_{m}-p_{j}{∥}_{2}}\right|, \end{array}$$

where $C_{i}^{\prime \prime }$ is the filtered subset of $S_{ij}$, *i* denotes the current iteration, α represents the angular consistency metric, and β is the adaptive angle constraint threshold derived from the distance constraint τ.

Finally, in the third stage, we employ standard RANSAC on $C^{\prime \prime }$ to obtain the final consensus set $C^{\prime \prime \prime }$ as the correct correspondence set. For keypoints in the filtered correspondence set, we use SVD decomposition to obtain the initial pose.

### 3.3. Pose Refinement and Local Registration

The pose estimated from local feature matching serves as a coarse alignment but can be inaccurate due to monocular ToF depth noise and non-uniform sampling, which degrade feature distinctiveness on the smooth aircraft surface.

To refine this coarse alignment, we adopt GICP instead of standard ICP or NDT [38]. GICP models local surface geometry using per-point covariance matrices, yielding a probabilistic plane-to-plane metric that is more robust to anisotropic ToF noise.

The registration problem is formulated as estimating a rigid-body transformation T∈SE(3) that minimizes the Mahalanobis distance between corresponding points in the source cloud $P=\{p_{i}\}$ and the target cloud $Q=\{q_{i}\}$:

(22) $$\begin{array}{c} T^{*}=\underset{T}{\arg\min}\sum_{i}d_{i}^{⊤}{\left(C_{i}^{Q}+TC_{i}^{P}T^{⊤}\right)}^{-1}d_{i}, \end{array}$$

where $d_{i}=q_{i}-Tp_{i}$ is the residual vector, and $C_{i}^{P}$ and $C_{i}^{Q}$ are the local covariance matrices around $p_{i}$ and $q_{i}$, respectively. Solving Equation (22) requires an iterative non-linear least squares method (e.g., Gauss-Newton) with an initialization $T_{\mathrm{init}}$. In this work, we use the following:

1. Initial refinement (keyframe): $T_{\mathrm{init}}\leftarrow T_{\mathrm{coarse}}$, where $T_{\mathrm{coarse}}$ is the coarse pose estimated from feature matching.
2. Temporal tracking (non-keyframe): $T_{\mathrm{init}}\leftarrow T_{t-1}$, where $T_{t-1}$ is the optimized pose from the previous frame.

### 3.4. Calibration

In most cases, we aim to obtain the pose change of the model in its own model coordinate system, namely $T_{M^{\prime }}^{M}$. Therefore, it is necessary to transform the computed $T_{reg}$ through Equation (9). This requires obtaining the transformation $T_{M}^{C}$ from model coordinate system *M* to camera coordinate system *C* at time 0. In our framework, $T_{M}^{C}$ is obtained by applying the aforementioned global registration procedure to register the CAD model (in *M*) with the time 0 point cloud (in *C*).

The model coordinate system uses the right-hand body axis convention with origin at the aircraft center of gravity: *x*-axis points along the fuselage toward the nose, *y*-axis points toward the right wing, and *z*-axis points toward the fuselage bottom. In practical measurement, CAD models of measurement targets are readily available. Therefore, registering the model point cloud in the camera coordinate system acquired at time 0 with the CAD model of the measurement target yields $T_{M}^{C}$.

Furthermore, to obtain the model’s pose change in the wind tunnel coordinate system $T_{W}$ or in the mechanism coordinate system $T_{Z^{\prime }}^{Z}$, one only needs to calibrate the transformation $T_{W}^{C}$ between the camera coordinate system and wind tunnel coordinate system *W* at time 0, or the transformation $T_{Z}^{C}$ with mechanism coordinate system *Z*.

## 4. Experiments and Results

### 4.1. Experimental Setup

#### 4.1.1. Datasets

Two datasets are used for performance evaluation: a simulated dataset and a real-world dataset.

The simulated dataset was generated in Blensor [39] by rendering noiseless single-view point clouds and subsequently introducing three types of synthetic noise: Gaussian noise, flying point noise, and simulated multipath interference (MPI). Specifically, 1% of the points were displaced by 5 pr to simulate flying points, where pr is point cloud resolution (the average distance between adjacent points in the point cloud). MPI was emulated by offsetting points along the sensor-to-point ray direction with a delay distance equal to 15% of the original range, blending the original and delayed distances with weights of 60–80% and 20–40%, respectively. This was followed by the addition of Gaussian noise with an intensity of 0.5 pr.

**Figure 4:**
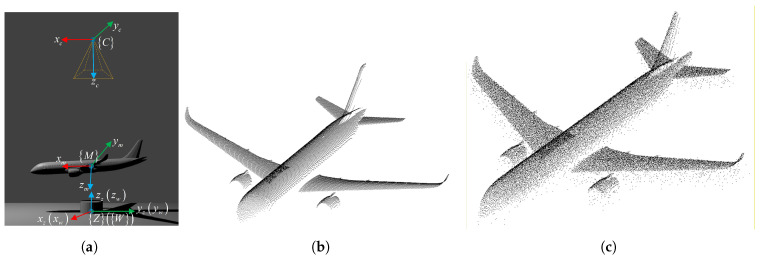
(**a**) Simulated scene, (**b**) acquired noiseless point cloud, and (**c**) point cloud with three types of simulated noise.

To simulate the wind tunnel mechanism, a cylinder with a radius of 1 m and a height of 1 m was placed in the world coordinate system. An aircraft model resembling the C919, with dimensions of 5.16 m × 5.03 m × 1.49 m, was rigidly attached to the cylinder. The attachment incorporated a $3^{\circ }$ rotation defined by XYZ Euler angles and a 2 m translation along the world *z*-axis. The ToF camera was positioned at world coordinates (0,0,8) m with a $2^{\circ }$ XYZ Euler misalignment to simulate installation error. The simulated scene and point clouds are shown in Figure 4.

We rotate the cylinder about its three axes individually with $5^{\circ }$ step size in the range $\left[-15^{\circ },15^{\circ }\right]$, acquiring 100 frames at each angle step to form the simulated dataset.

**Figure 5:**
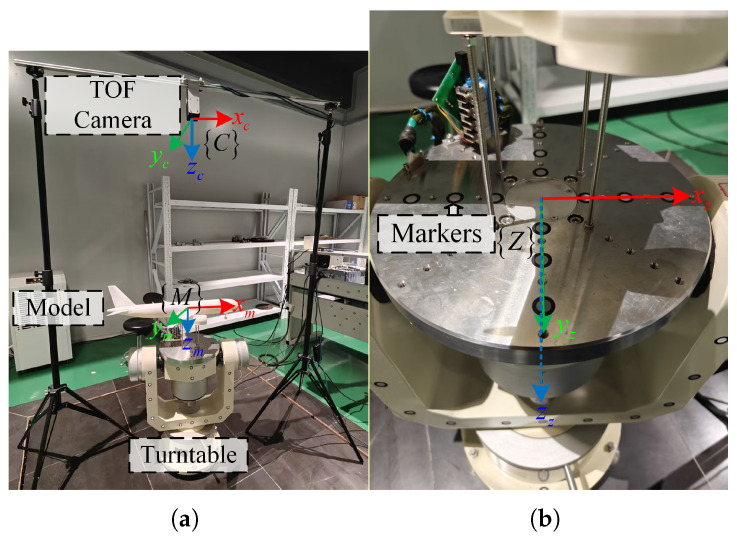
(**a**) Real experimental setup containing ToF camera, model, turntable, and computer; (**b**) attached fiducial points and defined turntable coordinate system.

To evaluate the proposed framework under realistic conditions, a real-world dataset was collected using the physical measurement platform shown in Figure 5, consisting of a ToF camera, a three-axis turntable, an aircraft model, and a computer. The camera was positioned approximately 1.2 m above the model. The ToF camera has a working distance of 8.3 m, a field of view of $69^{\circ }\times 54^{\circ }$, and a spatial resolution of 640×480 pixels. The aircraft model represents a generic passenger aircraft configuration (length 0.78 m, wingspan 0.71 m, and height 0.24 m).

**Figure 6:**
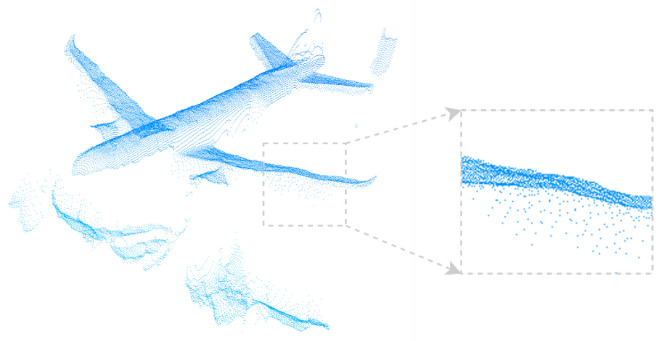
Model point clouds acquired from our custom real-world wind tunnel simulation platform.

The ToF camera and turntable are connected to the computer for data acquisition. To ensure consistency with the simulation experiments, the aircraft model was rotated around three axes (pitch, yaw, and roll) ranging from $-15^{\circ }$ to $15^{\circ }$ with a step size of $5^{\circ }$. At each step, 100 frames of point cloud data were collected to ensure statistical reliability. The ground truth poses were provided by the high-precision turntable. In the real experimental setup, the physical model is manufactured using rigid material and rigidly mounted on the turntable mechanism. Due to single-view acquisition and surface reflectance variations, the captured point clouds mainly cover the upper fuselage, wings, and part of the empennage, which is consistent with typical wind tunnel measurement scenarios. Figure 6 shows the model point clouds acquired by the ToF camera. Compared to simulated noise, the real-world acquired model point clouds exhibit more noise due to environmental factors and model surface material properties, including partial shape deficiencies and even local deformations.

It should be noted that in the real experiment, the transformation $T_{Z}^{C}$ from camera coordinate system to mechanism coordinate system *Z* at time 0 and the transformation from model coordinate system *M* to mechanism coordinate system *Z* are both unknown.

#### 4.1.2. Implementation Details

Our framework and algorithms run on a computer configured with an Intel Core Ultra 7 255H CPU and 32 GB of memory. Our algorithms are implemented based on the Point Cloud Library (PCL) [40] with multi-threading acceleration but without GPU acceleration. The first frame is selected as the keyframe. The algorithm parameters used in our experiments are summarized in Table 2. To ensure the method’s applicability to general scenarios, scale-dependent parameters (such as downsampling grid size) are defined relative to the point cloud resolution (pr). This dynamic scaling allows the pipeline to adapt to varying working distances and sensor resolutions. The quantization bit depths (16 for density; 24 for height) were empirically selected to balance feature distinctiveness and computational efficiency across both tested aircraft models [22].

#### 4.1.3. Evaluation Metrics

Since similarity transformation preserves rotation angle while changing the rotation axis, for the rotation component, we first extract the rotation angle $\hat{\theta }$ and rotation axis $\hat{a}={\left[{\hat{a}}_{x},{\hat{a}}_{y},{\hat{a}}_{z}\right]}^{T}$ from R:

(23) $$\begin{array}{cc} \hat{\theta } & =\arccos\left(\frac{\mathrm{tr}(R)-1}{2}\right),\\ \left[\begin{array}{c} {\hat{a}}_{x}\\ {\hat{a}}_{y}\\ {\hat{a}}_{z} \end{array}\right] & =\frac{1}{2\sin\hat{\theta }}\left[\begin{array}{c} r_{32}-r_{23}\\ r_{13}-r_{31}\\ r_{21}-r_{12} \end{array}\right]. \end{array}$$

where tr(R) is the trace of R. We then compute errors $e_{\theta }$ and $e_{a}$ between estimated rotation angle $\hat{\theta }$ and rotation axis $\hat{a}$ and their ground truth values, as well as the translation error:

(24) $$\begin{array}{cc} e_{\theta } & =\left|\hat{\theta }-\theta \right|,\\ e_{a} & =\arccos\left(\left|\hat{a}\;\cdot \;a\right|\right),\\ e_{t} & ={∥\hat{t}-t∥}_{2}. \end{array}$$

### 4.2. Accuracy Analysis

#### 4.2.1. Simulated Experiment Results

**Figure 7:**
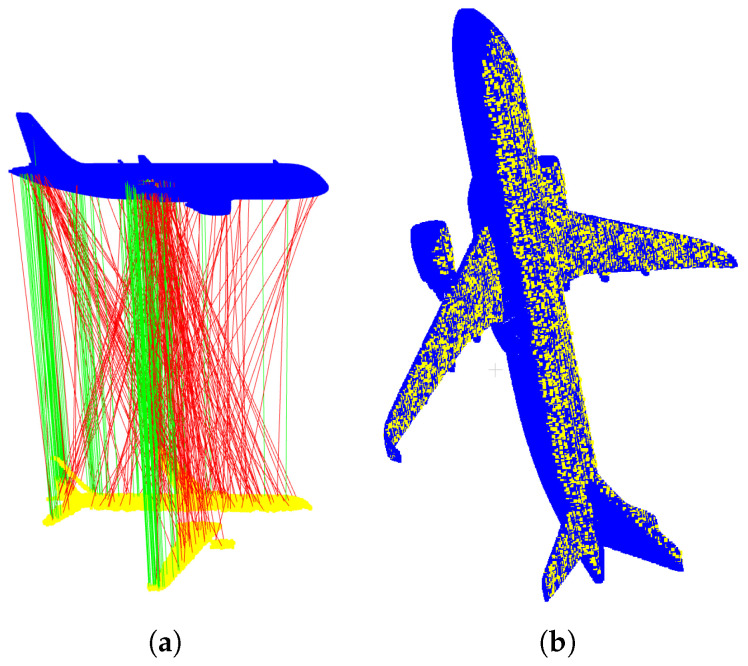
Registration results between model point cloud (yellow) acquired at time 0 and CAD model (blue): (**a**) initial correspondences (red) from feature matching and correct correspondences (yellow) after robust filtering; (**b**) camera point cloud after registration and CAD model.

Since coordinate system transformations are known in simulated experiments, we evaluate the final pose estimation error in the model coordinate system, where the influence of calibration errors is inherently included. The transformation $T_{M}^{C}$ from model coordinate system *M* to camera coordinate system *C* at time 0, as mentioned, is obtained through registration of the model with the acquired point cloud. Registration results are shown in Figure 7.

**Figure 8:**
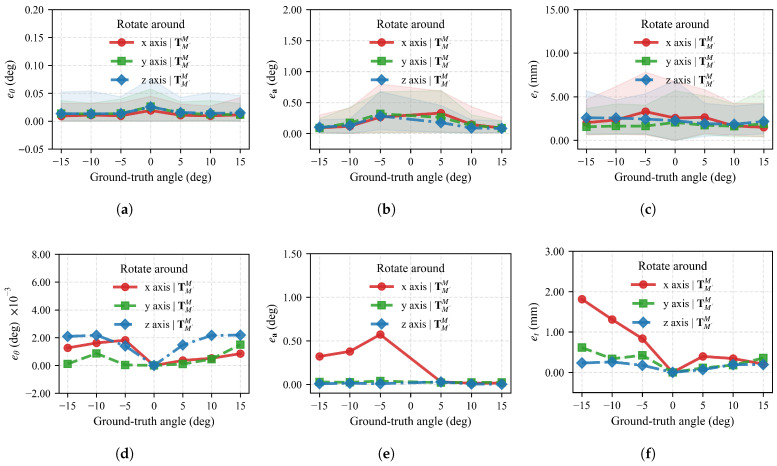
(**a**–**c**) show the rotation angle error $e_{\theta }$, rotation axis error $e_{a}$, and translation error $e_{t}$ under noiseless conditions, respectively. (**d**–**f**) show the same errors under noisy conditions. The *x*-axis represents ground truth rotation angles at different stages, with shaded regions indicating maximum and minimum values for each stage.

We compiled statistics for rotation angle error $e_{\theta }$ and translation error $e_{t}$ across all angles for different rotation axes, as shown in Figure 8 and Table 3. Results demonstrate that the proposed pose measurement method achieves high measurement precision and good stability. Under noiseless conditions, mean rotation angle errors for all three axes are below 1.60 × 10^−3∘^, with standard deviations all being zero, indicating excellent convergence precision and repeatable measurement results in ideal conditions. Mean translation errors are at most 0.70 mm, with the standard deviations also being zero.

When noise is introduced, rotation angle error means increase to 0.01–$0.02^{\circ }$, which are below $0.03^{\circ }$, with standard deviations around $7.70\times 10^{-3}$–$0.01^{\circ }$, indicating some measurement variation but overall being controllable. Translation error mean increase to 1.73–2.29 mm with standard deviations around 0.82–1.10 mm, showing moderate variation relative to means. Overall, the algorithm maintains good measurement precision and stability under noisy conditions, demonstrating strong robustness to the added point cloud noise.

#### 4.2.2. Real Experiment Results

In real experiments, transformations between coordinate systems are unknown except $T_{M}^{C}$ at time 0, which can be obtained through model–point cloud registration. However, both $T_{M}^{Z}$ from the model to mechanism coordinate system and $T_{C}^{Z}$ from the camera to mechanism coordinate system require additional calibration. We calibrate $T_{C}^{Z}$ and evaluate $T_{reg}$ after similarity transformation to the mechanism coordinate system.

**Figure 9:**
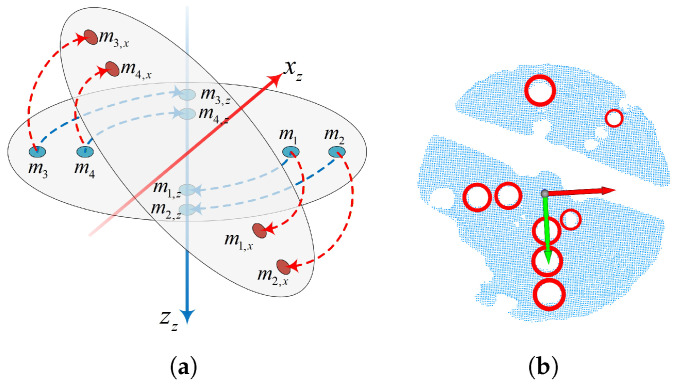
Turntable coordinate system obtained through fiducial point fitting. (**a**) Rotation illustration. (**b**) Example of a real-world acquired turntable surface, where red circles are detected fiducial points, the red arrow is the constructed turntable coordinate system’s *x*-axis, and the green arrow is the *y*-axis.

Since turntable rotary tables typically have high-precision threaded holes drilled by manufacturers, we attach fiducial points to these holes. The turntable rotates to *n* positions about the *z*-axis and *x*-axis; at each position, the ToF camera acquires turntable surface images, fitting fiducial points using established algorithms. Through circular arc fitting, we obtain the $x_{z}$ and $z_{z}$ axes of the turntable coordinate system; $y_{z}$ is obtained through cross product, as shown in Figure 9.

**Figure 10:**
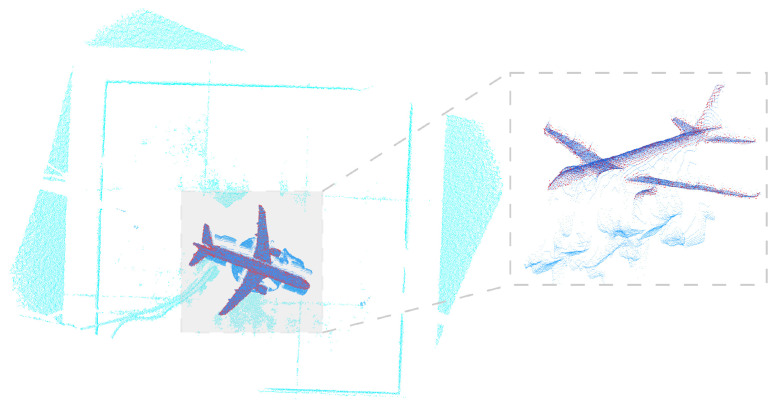
Keyframe point cloud example. Red points represent the registered model point cloud.

Successful keyframe registration scenarios and example ToF-acquired model point clouds are shown in Figure 10. Compared to simulated point clouds, real point clouds contain substantially more noise.

**Figure 11:**
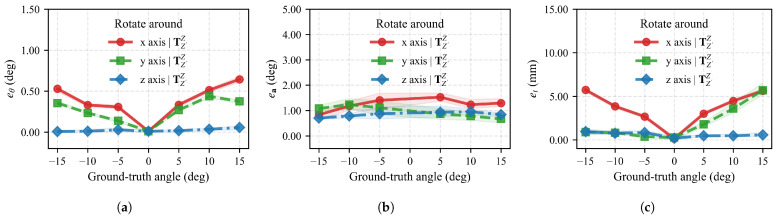
(**a**–**c**) show rotation angle error $e_{\theta }$, rotation axis error $e_{a}$, and translation error $e_{t}$, respectively. The *x*-axis represents ground truth rotation angles at different stages, with shaded regions indicating maximum and minimum values for each stage.

Rotation angle error $e_{\theta }$ and translation error $e_{t}$ across all angles for different axes are shown in Figure 11. Unlike simulated results, rotation around the *z*-axis yields the smallest error, while rotations around the *x* and *y* axes show rotation angle and translation errors increasing with rotation angle. Since model rotation around the *z*-axis approximates rotation around the camera optical axis, we term this phenomenon “axis sensitivity.” We analyze the causes of axis sensitivity in the Section 5.

Mean rotation angle error and standard deviation of the mean across all rotation angles are presented in Table 4. For rotation around the *z*-axis, the mean rotation angle deviation is $0.02^{\circ }$. In contrast, for model rotations around *x* and *y* axes, mean rotation angle errors reach $0.38^{\circ }$ and $0.26^{\circ }$, respectively. Standard deviations for rotations around all three axes are 0.01°.

**Figure 12:**
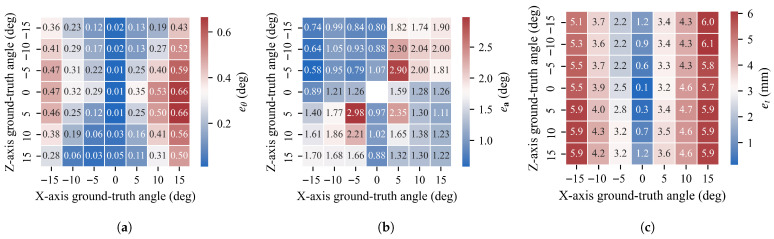
(**a**–**c**) show rotation angle error $e_{\theta }$, rotation axis error $e_{a}$, and translation error $e_{t}$ when simultaneously rotating about *z* and *x* axes. The *x*-axis represents the rotation angle around the *x*-axis, and the *y*-axis represents the rotation angle around the *z*-axis.

To further analyze real-world effectiveness with multi-axis rotations, we calculated poses when the turntable rotates simultaneously about *z* and *x* axes at different angles, with results in Figure 12. As rotation angles increase around the *x*-axis, both rotation angle error and translation error increase, while rotation around the *z*-axis maintains relatively consistent errors when the *x*-axis rotation angle is fixed. Minimum rotation angle error across all stages is only $0.01^{\circ }$, with a minimum translation error of only 0.1 mm.

Overall, the framework’s effectiveness is thoroughly validated, particularly achieving the highest precision when rotating around the *z*-axis.

### 4.3. Computational Efficiency

Our framework’s computational performance is summarized in Table 5. Real-world dataset input point clouds contain approximately 88,000 points, reduced to approximately 3800 points after preprocessing. Local feature construction is the most time-consuming step for keyframes. Total keyframe processing time, including a comprehensive global registration pipeline, is less than 120 ms. More importantly, all subsequent non-keyframe processing time is less than 25 ms, achieving pose output frame rates exceeding 40 FPS. Since global registration is executed only once on the initial frame, our framework easily enables real-time continuous pose measurement.

The computational cost is dominated by filtering and voxel downsampling, local feature construction and matching, and GICP-based local registration. When point density increases, the runtime of the nearest-neighbor search and correspondence evaluation typically grows rapidly if the raw point count is left uncontrolled. In our framework, voxel downsampling provides an explicit mechanism to bound the number of points, enabling an accuracy–speed trade-off by adjusting the grid size. For higher frame rates commonly required in large wind-tunnel facilities, the inter-frame motion is smaller, which generally benefits the convergence of local registration; therefore, the non-keyframe branch can maintain tracking as long as the per-frame point count is controlled. Finally, the main computational kernels (kNN search, descriptor computation, and GICP iterations) are amenable to parallelization; multi-threading is already used in our implementation, and further acceleration using SIMD/GPU or approximate nearest-neighbor structures can be adopted when higher resolution or >100 Hz update rates are required.

### 4.4. Comparison with Other Methods

Having validated our method’s performance, we further compare our proposed framework with mainstream methods in terms of accuracy, speed, and robustness. We selected several prevalent point cloud registration methods. Global registration methods include classical FPFH and recent MDCS [41] and TPSH [42], while local registration methods include classical ICP, GICP, and recent VGICP. FPFH uses PCL implementations, GICP and VGICP use author-provided source code, and TPSH and MDCS are implemented by us. All common parameters are kept consistent, with method-specific parameters using the original authors’ defaults. The characteristics of different local features are shown in Table 6.

Comparisons on keyframes from real-world experiments with model rotation around the *z*-axis are performed, computing each result 10 times and averaging, with results shown in Table 7.

The experimental results indicate that the proposed method achieves the highest registration accuracy, yielding the lowest errors across rotation angle, rotation axis, and translation metrics. In terms of efficiency, it ranks second overall, offering an optimal trade-off compared to competing methods. Specifically, while MDCS excels in feature construction speed (approx. 40% of ours), its matching process is significantly slower (approx. 3.9×) due to high feature dimensionality. Conversely, although FPFH achieves the fastest matching, its limited descriptive power results in substantial registration errors. Consequently, the proposed framework strikes the best balance between descriptive capability and computational cost, delivering superior precision with competitive real-time performance.

Local registration method comparison results are shown in Table 8. Initial values for each method are computed using our approach. GICP shows the shortest computation time and smallest registration error on our dataset, validating the reasonableness of our local registration method selection.

### 4.5. Evaluation of Different Geometric Shapes

**Figure 13:**
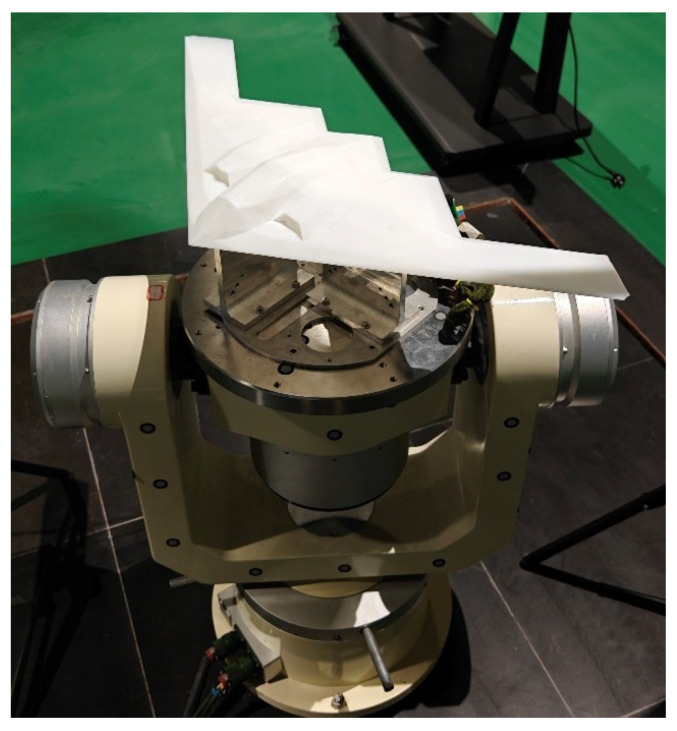
Model 2 used in real-world experiments.

To evaluate the generalization capability of the proposed framework across different objects, we introduced a second aircraft model (referred to as Model 2) in addition to the original model (Model 1, resembling the C919). As shown in Figure 13, Model 2 resembles a B-2 bomber configuration and exhibits a geometry significantly different from Model 1, particularly characterized by a flatter profile.

Experiments were conducted in an environment with a temperature of 22.4 °C and an illuminance of 11 Lux. Model 2 was rigidly mounted on the turntable and rotated around the *z*-axis to positions of $-15^{\circ }$, $0^{\circ }$, and $15^{\circ }$. For each angle, 100 frames were collected. The comparative results of rotation angle error, rotation axis error, and translation error for both models are presented in Table 9.

The mean rotation angle error for Model 2 across the three angles is $0.0349^{\circ }$, compared to $0.0249^{\circ }$ for Model 1, indicating a difference of approximately $0.01^{\circ }$. Both models exhibit the maximum rotation angle error at $15^{\circ }$. The rotation axis error remains approximately $1^{\circ }$. Although the translation error for Model 2 is slightly larger than that of Model 1, the error trends are consistent: errors are minimal at $0^{\circ }$ and increase as the rotation angle increases.

The mean and standard deviation values for Model 2 are generally higher than those for Model 1. This can be attributed to variations in geometric observability. Since Model 2 exhibits a “flatter” profile along the *z*-axis, the eigenvalues of its Hessian matrix in that direction are reduced. This implies that the optimization landscape becomes flatter, making the pose estimation more susceptible to noise and resulting in higher uncertainty. Nevertheless, the algorithm maintains a high level of accuracy for both geometries.

### 4.6. Evaluation of Real-World Environmental Factors

To assess the robustness of the algorithm under varying environmental conditions, we analyzed the effects of ambient illuminance and temperature.

#### 4.6.1. Impact of Ambient Illuminance

**Figure 14:**
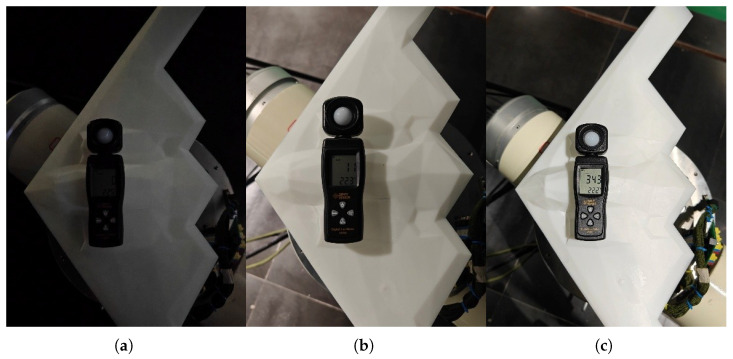
Real experimental scenarios under different ambient illuminance levels. (**a**–**c**) correspond to 0 Lux, 11 Lux, and 343 Lux, respectively.

First, with the environmental temperature controlled at 22.4 ± 0.2 °C, experiments were conducted under three different illuminance levels: 0 Lux (dark), 11 Lux (dim), and 343 Lux (bright), as shown in Figure 14.

Model 2 was rotated to $-15^{\circ }$ around the *z*-axis. The pose errors under different lighting conditions are summarized in Table 10. The results indicate that the three error metrics remain consistent across different illuminance levels. This robustness is attributed to the ToF camera operating in the near-infrared (NIR) spectrum (850 nm), which mitigates interference from visible light. This characteristic makes the system highly adaptable to indoor environments with controllable lighting, such as wind tunnels.

#### 4.6.2. Impact of Temperature and Drift Analysis

Next, under a constant illuminance of 11 Lux, we evaluated the performance at two different environmental temperatures: 16.4 °C and 22.4 °C. Model 2 was rotated to $-15^{\circ }$ around the *z*-axis. The results are shown in Table 11.

The errors remain consistent across different temperatures. ToF ranging errors are primarily influenced by internal sensor heat generation rather than ambient temperature. Ambient temperature mainly affects the heat dissipation efficiency. Once the ToF camera operates for a warm-up period, its internal temperature stabilizes, and the internal compensation mechanism ensures stable distance measurements, thereby maintaining registration accuracy.

**Figure 15:**
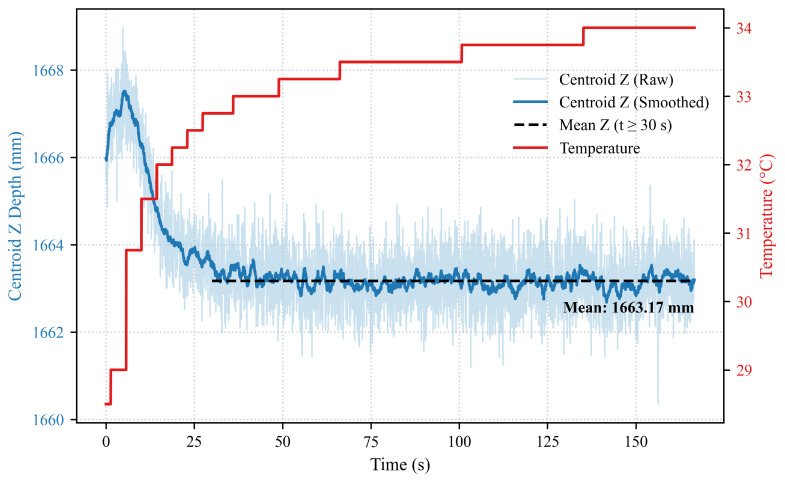
Scene centroid distance and ToF camera internal temperature variation curve with acquisition time.

To verify this, we monitored the *z*-axis depth of the scene centroid over a 3-min period, as depth stability is critical for point cloud generation. The relationship between the measured distance and the internal temperature of the camera over time is illustrated in Figure 15.

At the beginning of data collection, the internal temperature of the ToF camera rises rapidly, accompanied by an increase in the measured distance. Subsequently, the internal compensation mechanism activates, and the measured distance gradually stabilizes. As shown in the data, the centroid distance tends to reach equilibrium after approximately 30 s. Table 12 presents the stable centroid distances (t≥30 s) under different environmental and initial internal temperatures.

It can be observed that under different environmental temperatures but similar initial internal temperatures, the difference in mean distance is minimal (e.g., 0.03 mm). However, variations in internal temperature significantly affect the distance measurement (exceeding 0.1 mm). This confirms that the ranging error is dominated by internal thermal drift. It should be noted that all experimental data reported in this paper were collected after the device had reached thermal equilibrium (warm-up period > 30 s).

## 5. Error Analysis and Discussion

### 5.1. Axis-Sensitivity Analysis

In real experiments, we observed that when the model rotation axis approximately aligns with the camera optical axis—in our experiments, the turntable *z*-axis approximately aligns with the camera $z_{c}$ axis—registration precision significantly outperforms rotations where the axis is approximately perpendicular to the optical axis, namely turntable X/Y axis rotations. This “axis sensitivity” results from the combined effects of single-view geometric observability differences and anisotropic ToF depth measurement noise.

Taking point-to-plane ICP and GICP’s equivalent quadratic approximation near convergence as an example, residuals are expressed as [17]:

(25) $$r_{i}\left(T\right)=n_{i}^{⊤}\left(Rp_{i}+t-q_{i}\right),$$

with objective function $J\left(T\right)=\sum_{i}r_{i}{\left(T\right)}^{2}$. Here, $r_{i}$ denotes the residual for point pair *i*, $n_{i}$ represents the surface normal, R and t are the rotation and translation components, and $p_{i}$ and $q_{i}$ are corresponding points in the source and target clouds, respectively. Linearizing around convergence point $\hat{T}$ with pose perturbation δξ∈se(3), the Gauss-Newton Hessian approximation is $H\approx J^{⊤}WJ$, where W is the weight matrix, and J is the Jacobian of residuals with respect to pose perturbation. Censi provides a classical closed-form covariance estimation framework for ICP, emphasizing observability analysis under under-constrained conditions [43]. At a common first-order approximation, pose increment uncertainty is expressed as follows:

(26) $$\Sigma_{\delta \xi }\approx {\left(J^{⊤}WJ\right)}^{-1}\left(J^{⊤}W\;\Sigma_{sensor}\;WJ\right){\left(J^{⊤}WJ\right)}^{-1},$$

where $\Sigma_{sensor}$ characterizes point cloud measurement noise statistics, including depth noise, outliers, and quantization effects. From Equation (26), the spectral structure of $H=J^{⊤}WJ$—specifically its eigenvalue magnitudes and condition number—determines constraint strength for different degrees of freedom. Larger eigenvalues correspond to stronger constraints and smaller variance; conversely, flat valley bottoms result in high noise sensitivity and degeneracy, indicating weak observability.

ToF camera errors are not isotropic. Extensive surveys and evaluation work show that ToF depth measurement error along the range direction, approximately along $z_{c}$, typically significantly exceeds lateral error projected from pixel angular resolution to $x_{c}$ and $y_{c}$ planes [44]. This error varies with distance, incident angle, and reflectance and produces structured errors including flying pixels—also known as mixed pixels—and multipath interference arising from multiple reflections, commonly abbreviated as MPI.

When model rotation approximately aligns with the camera optical axis, the point cloud produces significant tangential displacement on the image plane, more constrained by lateral resolution and contour geometry, with weaker coupling to ToF depth noise. Therefore, H typically has larger eigenvalues in the rotation freedom direction, yielding smaller rotation uncertainty and more stable convergence. In contrast, rotation around $x_{c}$ or $y_{c}$ significantly changes many points’ depth values, while ToF depth noise and MPI, along with flying point error, are stronger in this direction, causing H eigenvalues to decrease and making rotation estimation more noise-sensitive. Additionally, single-view rotations around $x_{c}$ or $y_{c}$ more easily cause self-occlusion, reducing effective overlap and destabilizing correspondences.

Consequently, geometric observability—determined by visible surface and shape distribution—combined with ToF anisotropic noise, jointly determines estimation difficulty for different rotation axes. This provides guidance for wind tunnel camera placement: if experimental motion is primarily single-axis rotation, the rotation axis should approximate alignment with the camera optical axis; if unavoidable large rotations around $x_{c}$ or $y_{c}$ occur, multi-view coverage, increased overlap, or backend optimization are needed to compensate for weak observability directions.

### 5.2. Error Sources and Propagation

Final pose error in our framework results from the combined effects of sensor errors, registration errors, calibration errors, and systematic errors, propagating, coupling, and amplifying through coordinate transformation chains. At the sensor level, ToF point cloud measurement errors primarily stem from depth random noise and systematic bias, often exhibiting anisotropic distribution. At the algorithmic level, coarse registration suffers from noise and missing data, reducing local feature discriminability, causing initial value bias; fine registration more easily falls into local minima or exhibits unstable convergence in weakly observable directions. At the calibration level, CAD models are ideal, complete geometric models, while actual ToF point clouds are noisy, incomplete, single-view observations, making the CAD–point cloud registration matrix $T_{M}^{C}$ prone to systematic bias. Additionally, the camera–mechanism calibration matrix $T_{C}^{Z}$ relies on fitting limited, spatially constrained fiducial points, with precision bounded by point cloud quality and geometric conditioning, introducing inherent errors in mechanism coordinate system pose and origin. Furthermore, at the system level, temperature drift and model micro-vibrations during experiments effectively manifest as pose disturbances.

To uniformly describe these errors and analyze propagation, we employ the Lie group SE(3) multiplicative perturbation model, where SE(3) denotes the special Euclidean group representing rigid body transformations. For any pose T∈SE(3), the nominal value is $\bar{T}$, and the perturbation is δξ∈se(3), where se(3) denotes the Lie algebra representing infinitesimal motion. Using the left perturbation model from Equation (10), and since $T_{Z}^{C}$ is the inverse of $T_{C}^{Z}$, we express the first two terms as left perturbations:

(27) $$\begin{array}{cc} T_{C}^{Z} & \approx \exp\left(\delta \xi_{C}^{Z}\right)\;\cdot \;{\bar{T}}_{C}^{Z},\\ T_{reg} & \approx \exp\left(\delta \xi_{reg}\right)\;\cdot \;{\bar{T}}_{reg},\\ T_{Z}^{C} & \approx {\bar{T}}_{Z}^{C}\;\cdot \;\exp\left(-\delta \xi_{C}^{Z}\right). \end{array}$$

Let $T_{Z^{\prime }}^{Z}\approx T_{C}^{Z}T_{reg}T_{Z}^{C}$. At first-order approximation,

(28) $$\delta \xi_{Z^{\prime }}^{Z}\approx \left(I-{\mathrm{Ad}}_{{\bar{T}}_{Z^{\prime }}^{Z}}\right)\delta \xi_{C}^{Z}+{\mathrm{Ad}}_{{\bar{T}}_{C}^{Z}}\delta \xi_{reg},$$

where ${\mathrm{Ad}}_{T}$ denotes the adjoint representation of transformation T, which describes how perturbations transform between coordinate frames. Assuming independence of two perturbations, covariance propagation is as follows:

(29) $$\Sigma_{\delta \xi_{Z^{\prime }}^{Z}}\approx A\;\Sigma_{\delta \xi_{C}^{Z}}\;A^{⊤}+{\mathrm{Ad}}_{{\bar{T}}_{C}^{Z}}\;\Sigma_{\delta \xi_{reg}}\;{\mathrm{Ad}}_{{\bar{T}}_{C}^{Z}}^{⊤},$$

where $A=I-{\mathrm{Ad}}_{{\bar{T}}_{Z^{\prime }}^{Z}}$. Equations (28) and (29) show that calibration and registration uncertainties couple to downstream coordinate systems through similarity transformation. In particular, when the camera and model have large translation and rotation perturbations, they are significantly coupled to translation errors.

In this work, we use only the first frame as a keyframe for global registration and perform local tracking for subsequent frames. This design is justified by the smooth-motion assumption in typical wind-tunnel tests, which limits inter-frame pose changes and enables stable convergence of local registration. For longer experiments or cases with larger pose variations (e.g., abrupt motion, severe occlusion, or drift accumulated over time), relying on a single initial keyframe may increase the risk of tracking degradation and local-minimum convergence. In such situations, the framework can be extended by introducing an adaptive keyframe strategy, e.g., triggering re-initialization when the registration residual, overlap ratio, or inlier count indicates reduced alignment quality, or by periodically inserting keyframes at a fixed interval. These mechanisms would improve long-term robustness at the expense of occasional increases in latency.

## 6. Conclusions

This paper addresses pose measurement for aircraft in wind tunnels, proposing and implementing an efficient measurement framework fusing keyframe global feature matching with non-keyframe local fine registration. By analyzing ToF camera imaging models and aircraft motion constraints, we design a complete pipeline including point cloud preprocessing, global coarse registration based on improved hand-crafted features, and local fine registration based on temporal continuity. Experiments and error analysis demonstrate that while ensuring real-time output, the system achieves rotation angle errors as low as $0.02^{\circ }$ and translation errors of 0.61 mm at over 40 FPS, demonstrating accurate and real-time pose measurement.

The research reveals that constrained by geometric constraints and depth noise coupling, the system exhibits pronounced axis sensitivity, where rotation precision around the optical axis (*z*-axis) significantly exceeds rotations around horizontal axes (x/y-axes). This finding provides theoretical reference for wind tunnel camera placement: model rotation axes should approximate alignment with the camera optical axis.

Future work will focus on three main directions:

1. Extended real-world validation and generalization: The proposed framework will be further validated in operational wind tunnel environments through long-duration experiments under airflow-induced vibration and environmental disturbances. In addition, its generalization capability will be evaluated on aircraft models with different geometries, scales, and surface characteristics to assess robustness beyond a single model.
2. Robustness under high-dynamic and extreme motion conditions: While the current framework targets typical wind tunnel motion patterns, future studies will investigate its performance under more challenging scenarios, including rapid rotations, high-frequency vibrations, and transient disturbances, to better characterize system limits and error behavior.
3. System-level comparison and performance scaling: Future work will include direct comparisons with multi-camera photogrammetry-based pose measurement systems under identical experimental setups, as well as GPU-accelerated and parallel implementations of key modules to enable higher frame rates (e.g., >100 FPS) for high-speed measurement applications.

## Figures and Tables

**Table 1:** Comparison of pose measurement methods for wind tunnel experiments.

Method	Sensor	Typical Accuracy	Real-Time
IMU + Kalman Filtering [6]	IMU	Medium	High
RGB + PnP/Optimization [7]	RGB	High	Medium
RGB + Deep Learning [8]	RGB	Low-Medium	Medium
Structured light + Phase Shift [10]	Structured light	Very high	Low
LiDAR + ICP/NDT [11]	LiDAR	Medium	Medium
Proposed framework	ToF	High	High

**Table 2:** Algorithm parameter settings used in experiments.

Module	Parameter	Value
Preprocessing	Downsampling grid size	2 pr
Preprocessing	Statistical filter neighbors	50
Local feature	$M_{a}$	4
Local feature	$N_{r}$	4
Local feature	σ	0.7
Local feature	Density quantization bit depth	16
Local feature	Height quantization bit depth	24
TCF-RANSAC	Maximum iterations	1,000,000
GICP	Maximum iterations	50

**Table 3:** Rotation angle, rotation axis, and translation errors for different rotation axes under noiseless and noisy conditions in simulated experiments (mean ± std).

Rotation Axis	Error Type	With Noise	Noiseless
*x*-axis	$e_{\theta }$ (deg)	$0.01\pm 7.70\times 10^{-3}$	$9.00\times 10^{-4}\pm 1.27\times 10^{-9}$
	$e_{a}$ (deg)	0.17±0.10	$0.22\pm 2.70\times 10^{-7}$
	$e_{t}$ (mm)	2.29±1.10	$0.70\pm 3.35\times 10^{-6}$
*y*-axis	$e_{\theta }$ (deg)	$0.01\pm 8.70\times 10^{-3}$	$4.00\times 10^{-4}\pm 1.19\times 10^{-8}$
	$e_{a}$ (deg)	0.18±0.09	$0.03\pm 1.95\times 10^{-8}$
	$e_{t}$ (mm)	1.73±0.82	$0.29\pm 4.02\times 10^{-6}$
*z*-axis	$e_{\theta }$ (deg)	0.02±0.01	$1.60\times 10^{-3}\pm 1.36\times 10^{-9}$
	$e_{a}$ (deg)	0.14±0.06	$0.01\pm 1.96\times 10^{-7}$
	$e_{t}$ (mm)	2.25±1.02	$0.16\pm 1.54\times 10^{-6}$

**Table 4:** Mean rotation angle error, rotation axis error, and translation error for different rotation axes in real experiments (mean ± std).

Rotation Axis	$e_{\theta }$ (deg)	$e_{a}$ (deg)	$e_{t}$ (mm)
*x*-axis	0.38±0.01	1.24±0.08	3.64±0.08
*y*-axis	0.26±0.01	0.96±0.07	1.91±0.13
*z*-axis	0.02±0.01	0.85±0.05	0.61±0.08

**Table 5:** Computational performance for keyframe and non-keyframe processing on real-world datasets (mean ± std).

Processing Step	Keyframe (ms)	Non-Keyframe (ms)
Preprocessing	$14.70\pm 2.62\times 10^{-3}$	$14.50\pm 3.75\times 10^{-3}$
Keypoint extraction	$0.02\pm 2.23\times 10^{-6}$	–
Local feature construction	$89.50\pm 3.39\times 10^{-3}$	–
Feature matching	$3.90\pm 3.34\times 10^{-3}$	–
Pose refinement (local registration)	$5.97\pm 2.91\times 10^{-3}$	$9.75\pm 7.73\times 10^{-3}$
Total	$114.09\pm 5.48\times 10^{-3}$	$24.25\pm 8.60\times 10^{-3}$

**Table 6:** Comparison of local features.

Method	Based on LRF	Length
FPFH	No	33
TPSH	Yes	245
MDCS	Yes	177
Ours	Yes	48

**Table 7:** Comparison of global registration methods on real keyframes with model rotation around the *z*-axis (mean ± std).

Method	FPFH	TPSH	MDCS	Ours
Rotation angle error (deg)	0.33±0.26	0.22±0.19	0.32±0.17	0.20±0.10
Rotation axis error (deg)	1.91±0.94	1.55±0.76	1.00±0.41	0.98±0.51
Translation error (mm)	2.89±1.16	3.50±1.06	3.12±2.04	2.72±1.16
Local feature construction time (ms)	123.17±10.27	102.86±7.18	21.40±4.69	54.96±12.45
Feature matching time (ms)	1.65±1.16	4.44±2.50	9.99±0.98	2.54±1.89

**Table 8:** Comparison of local registration methods using initial poses estimated by the proposed framework (mean).

Method	ICP	VGICP	GICP
Rotation angle error (deg)	0.04	0.05	0.02
Rotation axis error (deg)	0.32	0.60	0.29
Translation error (mm)	0.81	1.12	0.71
Computation time (ms)	15.07	8.20	7.51

**Table 9:** Pose errors of models with different geometric shapes under identical conditions (mean ± std).

Ground Truth Rotation (deg)	−15		0		15	
Model	1	2	1	2	1	2
Rotation Angle Error (deg)	0.01 ± 0.01	0.03 ± 0.02	0.01 ± 0.45$\times 10^{-2}$	0.03 ± 0.02	0.06 ± 0.01	0.05 ± 0.04
Rotation Axis Error (deg)	0.70 ± 0.02	1.15 ± 0.03	/	/	0.84 ± 0.03	1.17 ± 0.13
Translation Error (mm)	0.94 ± 0.10	1.56 ± 0.05	0.19 ± 0.08	0.15 ± 0.06	0.57 ± 0.08	0.96 ± 0.17

**Table 10:** Pose error of the same model under different ambient illuminance (mean).

Ambient Illuminance (Lux)	0	11	343
Rotation angle error (deg)	0.04	0.03	0.04
Rotation axis error (deg)	1.13	1.15	1.15
Translation error (mm)	1.68	1.56	1.57

**Table 11:** Pose error of the same model under different ambient temperatures (mean).

Temperature (°C)	16.4	22.4
Rotation angle error (deg)	0.04	0.03
Rotation axis error (deg)	1.13	1.15
Translation error (mm)	1.67	1.56

**Table 12:** Stable centroid distance values of scenes under different ambient temperatures and the ToF camera’s internal temperature at the start of data collection (mean).

Ambient Temperature (°C)	Sensor Temperature (°C)	Distance (mm)
21.40	27.50	1663.23
20.30	27.50	1663.26
21.40	29.25	1663.44
21.40	28.50	1663.17

## Data Availability

Data underlying the results presented in this paper are not publicly available at this time but may be obtained from the authors upon reasonable request.
